# Supplementary material for: Proteome-wide analysis of USP14 substrates revealed its role in hepatosteatosis via stabilization of FASN
Source: Nat Commun. 2018 Nov 13;9:4770. doi: 10.1038/s41467-018-07185-y (PMC6233205; doi:10.1038/s41467-018-07185-y)
Supplement: Supplementary file 1 — Supplemental infomation [file 41467_2018_7185_MOESM1_ESM.pdf]

# Proteome-wide analysis of USP14 substrates revealed its role in hepatosteatosis via stabilization of FASN

Liu et al.

## Up-regulated USPs in livers from two groups of mice

| USPs | Fold Change | *P* Value |
|---|---|---|
| **Up-regulated (HFD / ND)** | | |
| **USP14** | **12.79** | **0.0013** |
| USP20 | 7.43 | 0.037 |
| USP11 | 6.82 | 0.0056 |
| USP2 | 3.65 | 0.018 |
| USP46 | 2.87 | 0.029 |
| **Down-regulated (HFD / ND)** | | |
| USP33 | 0.58 | 0.0025 |
| USP28 | 0.42 | 0.032 |

**Supplementary Figure 1** Dys-regulated USPs in livers from C57BL/6 mice fed a normal diet (ND) or high-fat-diet (HFD).

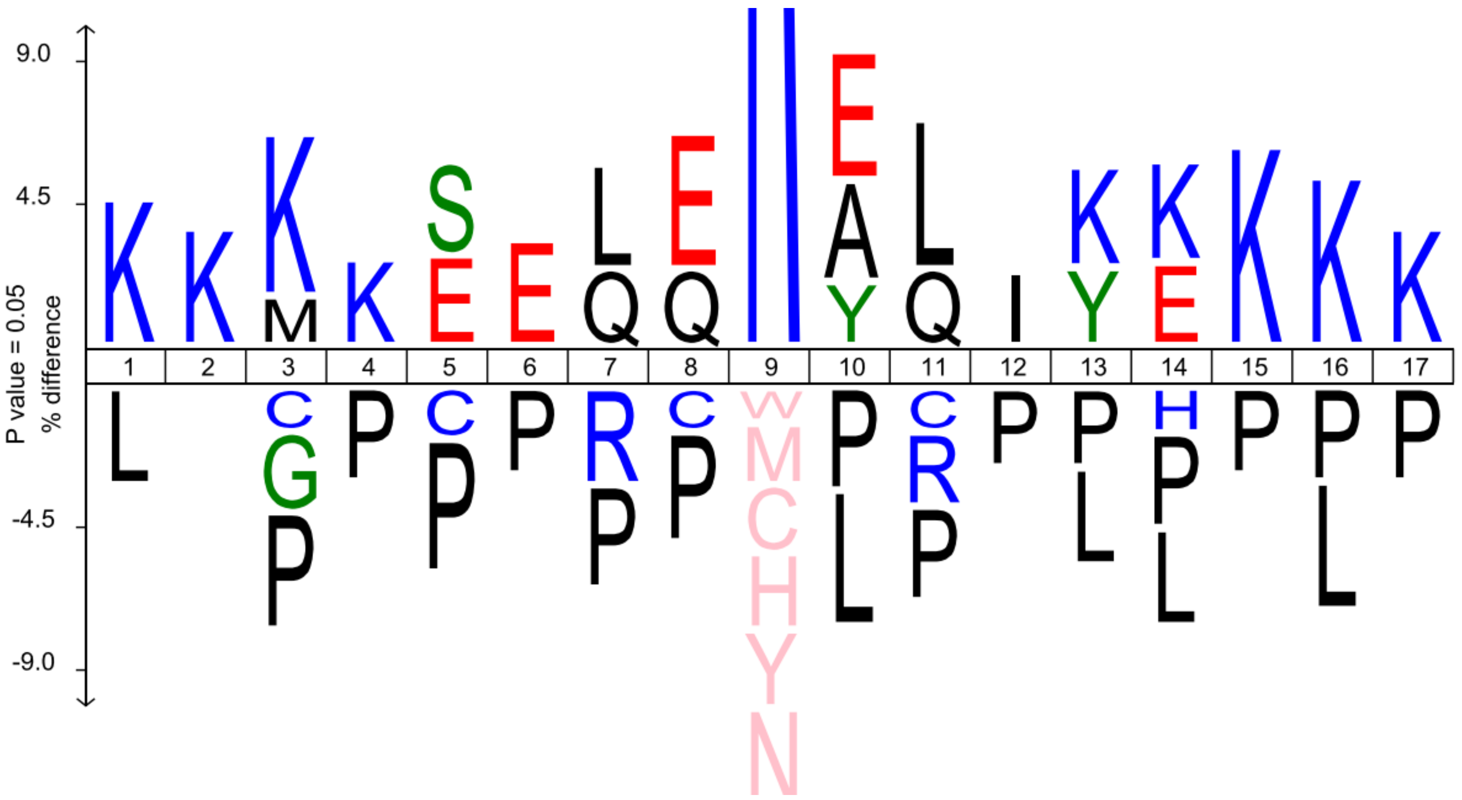


**Supplementary Figure 2** Motif analysis of up-regulated ubiquitin sites. The flanking sequences of up-regulated ubiquitin sites was analyzed using iceLogo software.

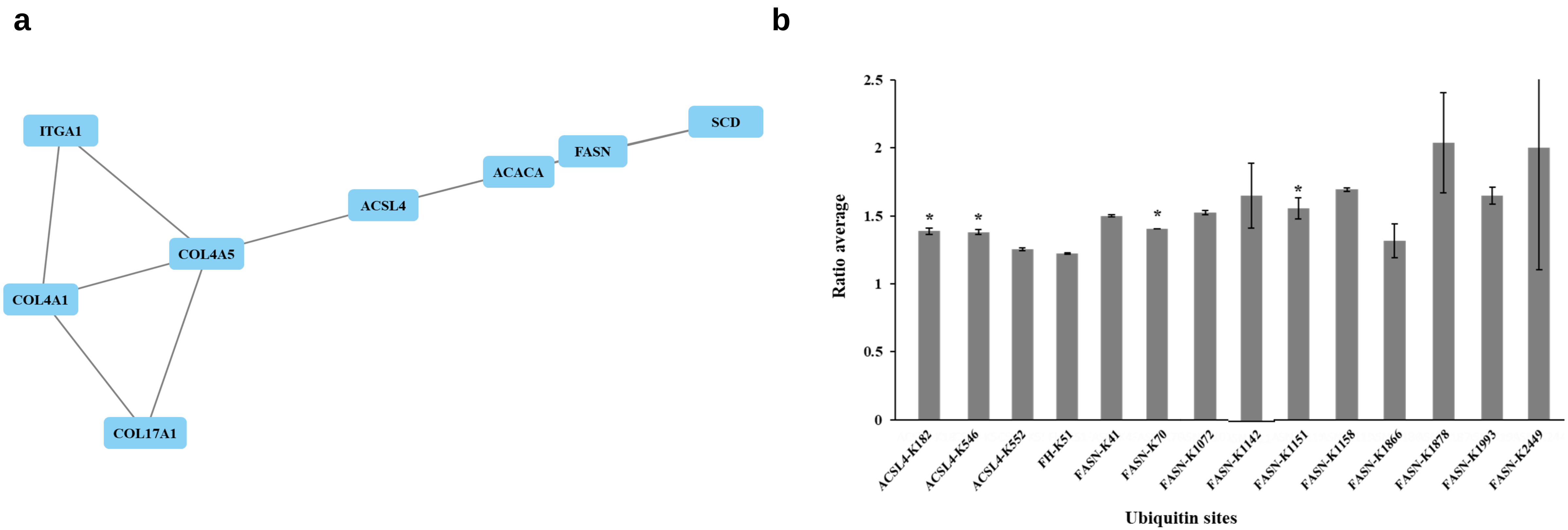


**Supplementary Figure 3** Bioinformatic analysis of significantly changed proteins and log2 ratio of several up-regulated ubiquitin sites. **a** The list of highly enriched clusters of up-regulated and down-regulated proteins identified in SILAC process. **b** Ratio of up-regulated ubiquitin sites of three key enzymes listed in Figure 3C in four replicates.

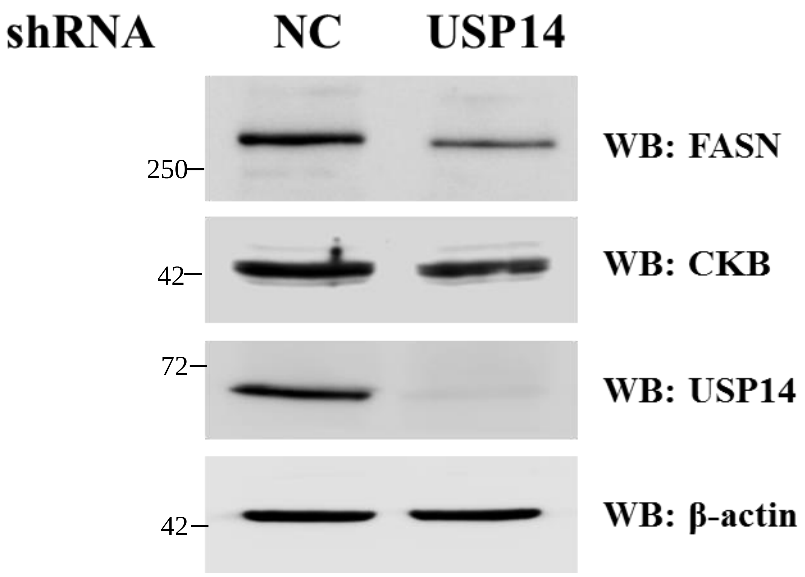


**Supplementary Figure 4** The lysate from HeLa cells stably expressing shRNAs against NC or USP14 were incubated with anti-FASN, anti-CKB, and anti-USP14 antibody. Their protein levels were detected by Western blot with the indicated antibodies.

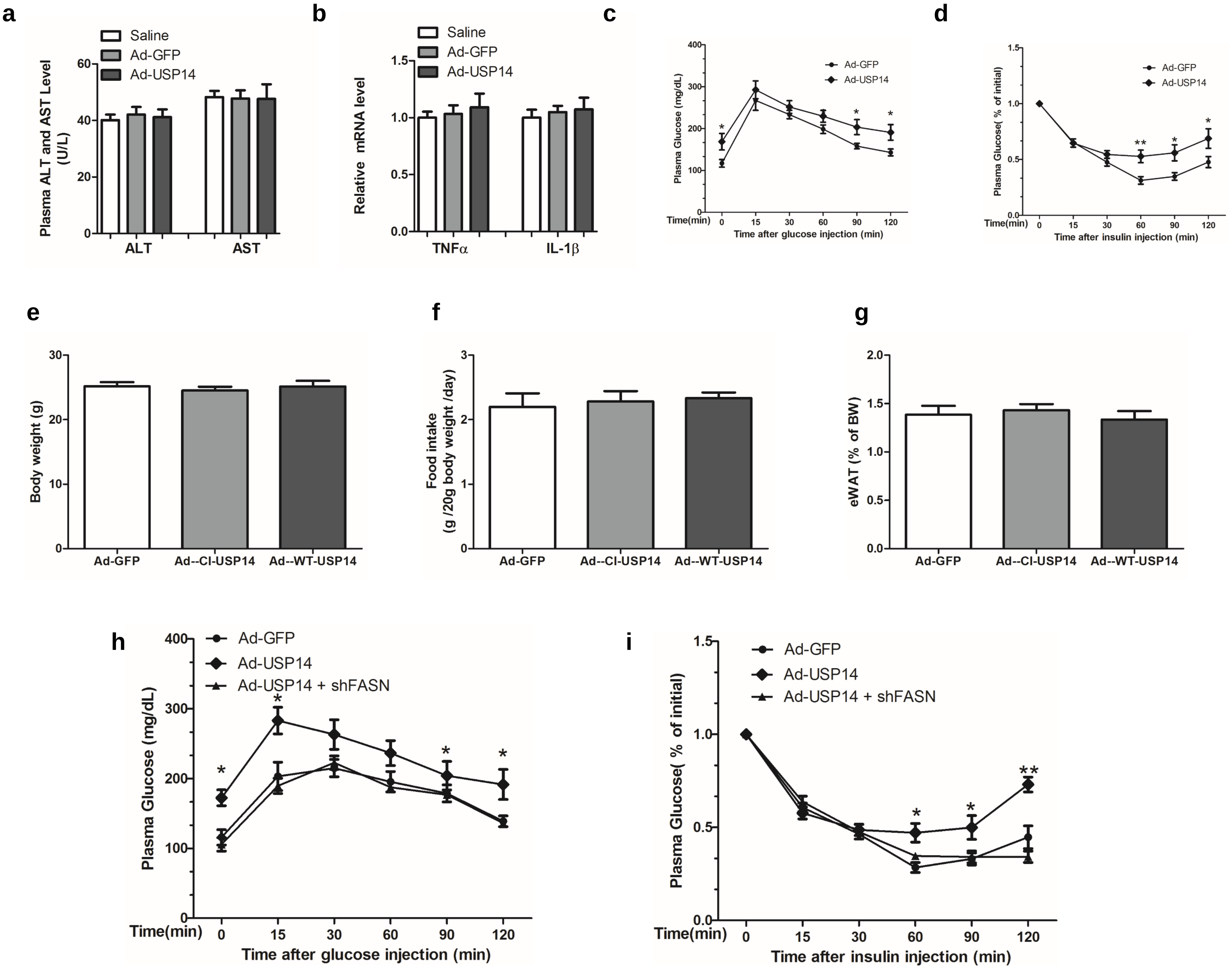


**Supplementary Figure 5 a,b** Plasma ALT, AST levels and expression of hepatic pro-inflammatory cytokines between Ad-USP14, Ad-GFP and saline treated C57BL/6 mice. **c,d** Glucose and insulin tolerance tests in C57BL/6 mice injected with Ad-GFP and Ad-USP14. **e,f,g** Body weight (**e**), food intake () and epididymal fat (EWAT) weight () **g** in C57BL/6 mice infected with Ad-WT-USP14, Ad-CI-USP14 or Ad**f**-GFP. **h,i** Glucose and insulin tolerance tests in Ad-GFP, Ad-USP14 and Ad-USP14+shFASN mice. Data are presented as mean ± SEM.

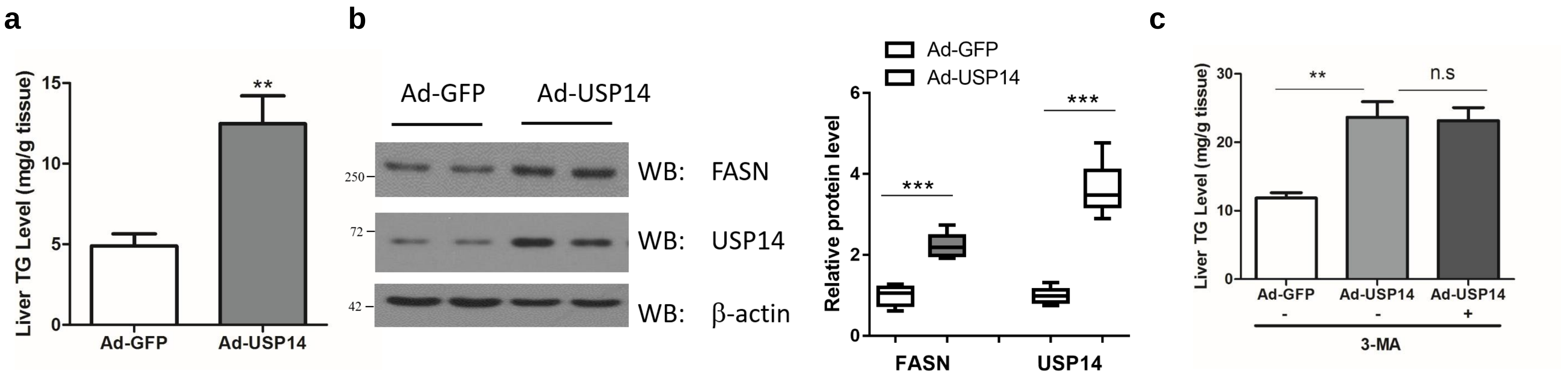


**Supplementary Figure 6 a** Live TG levels in C57BL/6 mice. Mice were treated with streptozotocin (STZ), which induced insulin deficiency due to the selective pancreatic beta-cell toxicity. 14 days later, mice were administrated with adenovirus containing USP14 or GFP via tail vein injection. **b** The protein levels of FASN, USP14 and b actin were detected by western blot with the indicated antibodies. A representative western blot was shown. The quantification plot was based on scanning densitometry analysis (n=6 for each group) using the ImageJ software (v 1.8.0). ***p < 0.001 versus indicated groups (Student's t-test). **c** Ad-GFP and Ad-USP14 mice were treated with 3-MA, an autophagy inhibitor. Live TG level were measured and shown in the bar chart.

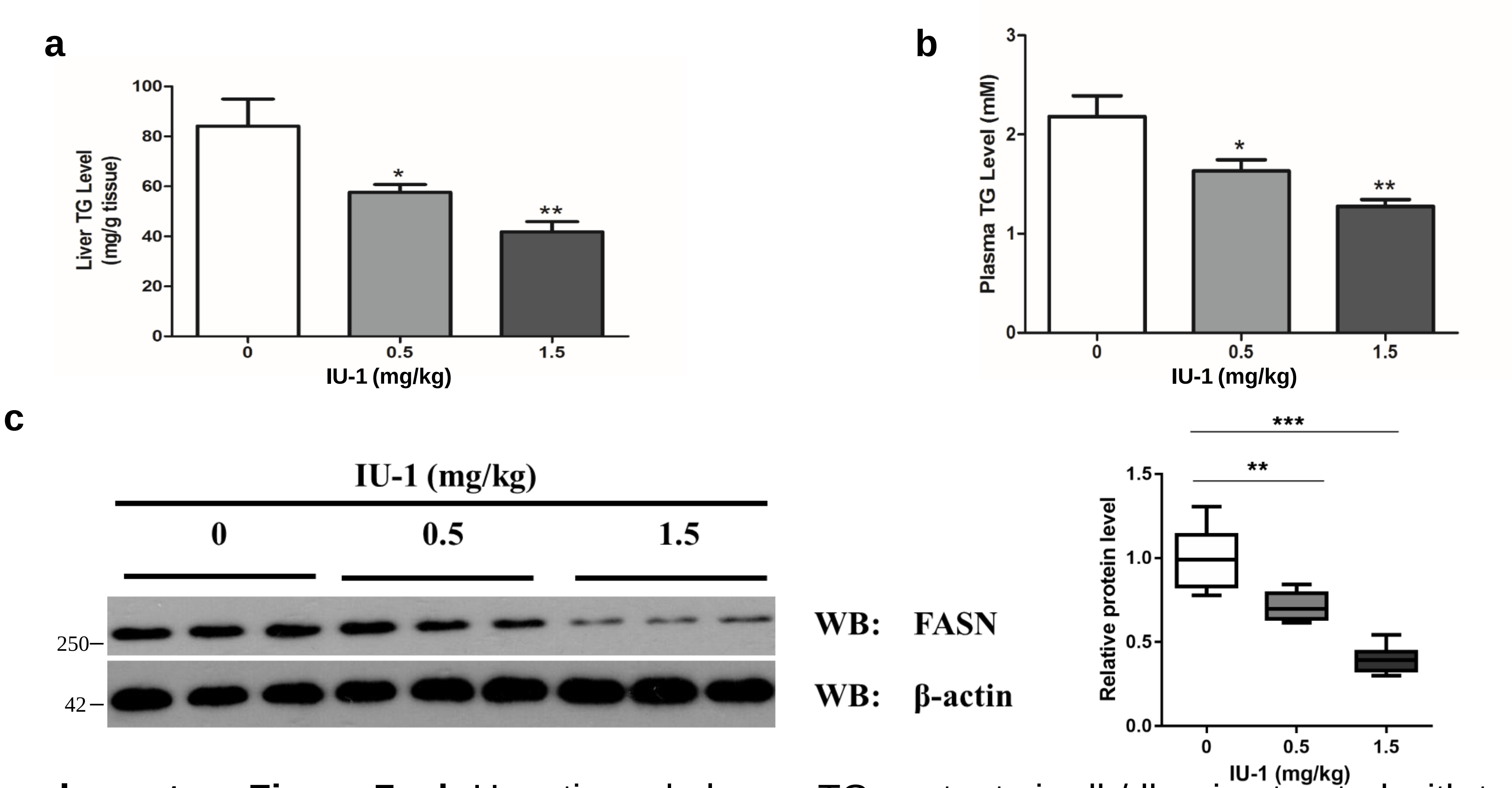


**Supplementary Figure 7 a,b** Hepatic and plasma TG contents in db/db mice treated with two doses of IU-1 or vehicle control for 7 days. Data are presented as mean ± SEM. *p < 0.05; **p < 0.01. **c** Representative protein levels of FASN in three groups of *db/db* mice. A representative western blot was shown. The quantification plot was based on scanning densitometry analysis (n=6 for each group). ***p < 0.001 versus indicated groups (Student's t-test).

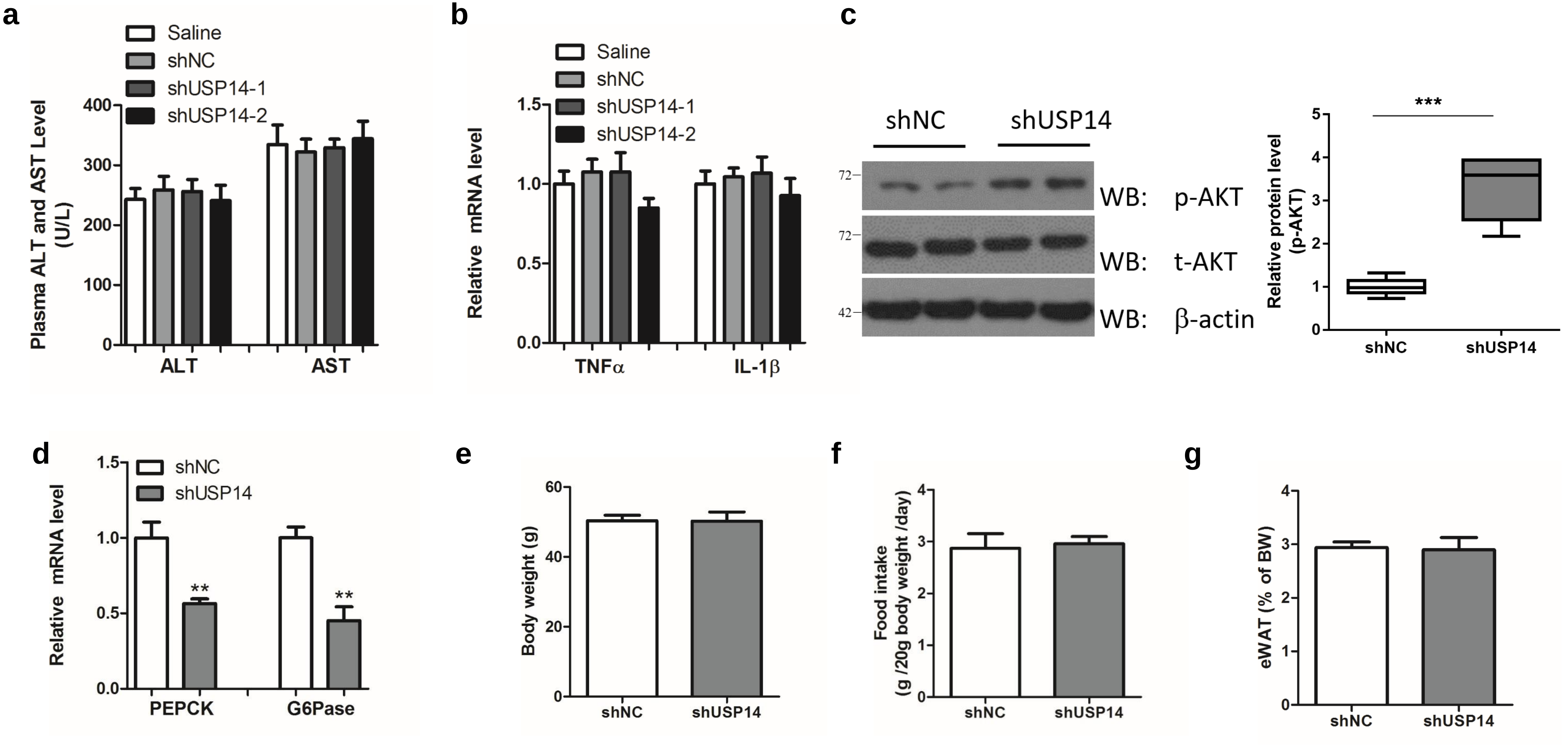


**Supplementary Figure 8 a,b** Plasma ALT, AST levels and expression of hepatic pro-inflammatory cytokines between shUSP14, shNC and saline treated *db/db* mice. **c,d,e,f,g** Representative phosphorylated AKT levels (Serine 473) in the livers. A representative western blot was shown. The quantification plot was based on scanning densitometry analysis (n=6 for each group). ***p < 0.001 versus indicated groups (Student's t-test) (**c**), Relative mRNA levels of hepatic gluconeogenic genes (PEPCK and G6Pase) (**d**), body weight (**e**), food intake (**f**) and epididymal fat (EWAT) weight (**g**) in *db/db* mice injected with adenoviral shUSP14 or shNC.

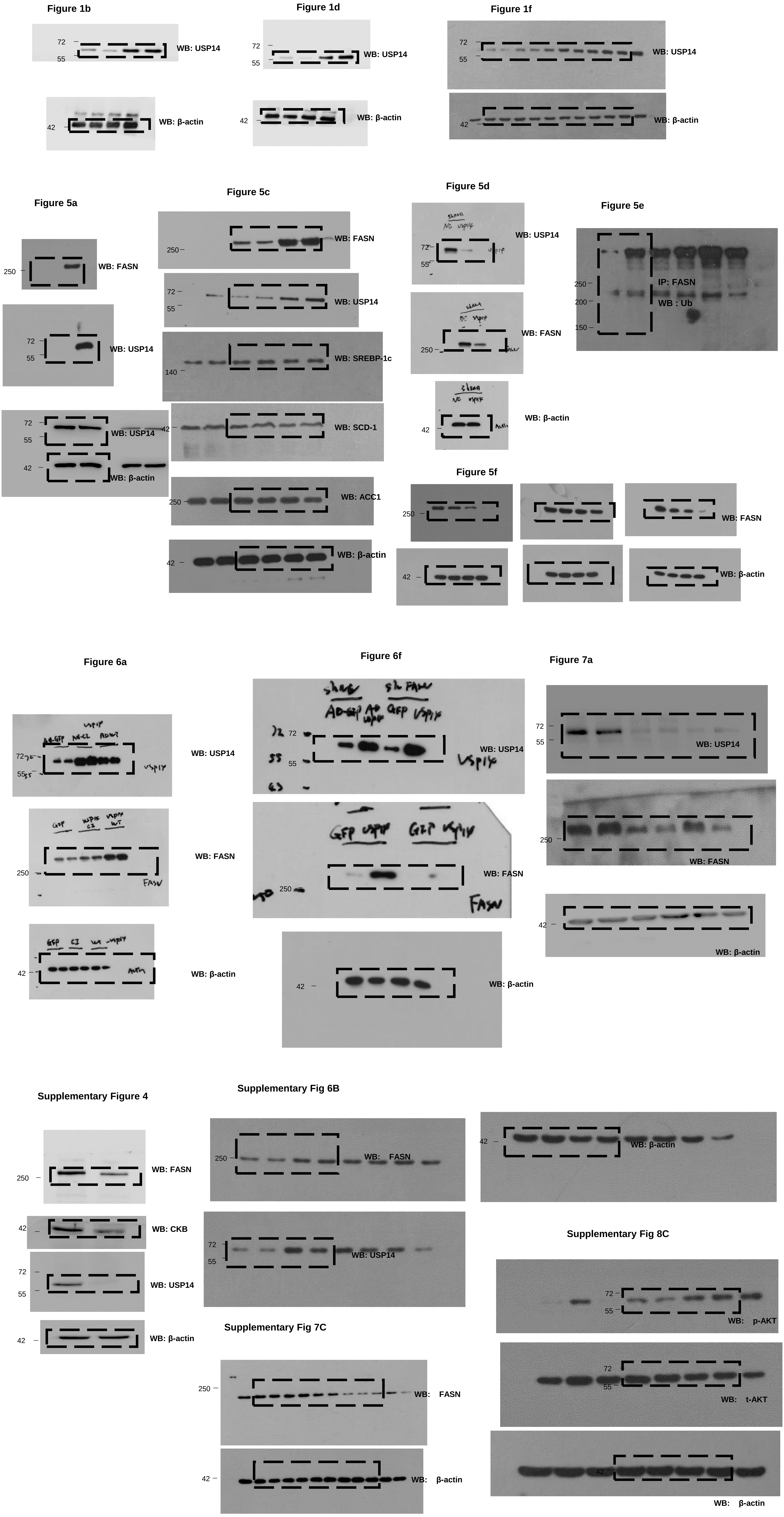


**Supplementary Fig. 9.** All the films for Western Blot analysis presented in this study with molecular weight markers.
