# Supplementary material for: Proteome-wide analysis of USP14 substrates revealed its role in hepatosteatosis via stabilization of FASN
Source: Nat Commun. 2018 Nov 13;9:4770. doi: 10.1038/s41467-018-07185-y (PMC6233205; doi:10.1038/s41467-018-07185-y)
Supplement: Supplementary file 3 — Description of Additional Supplementary Files [file 41467_2018_7185_MOESM3_ESM.pdf]

Supplementary Data 1.

List of significantly down- and up- regulated proteins in response to USP14KD in 4 replicates. p value < 0.05 and ≥1.2 fold-of-change was considered significant. NA: Not available for identification or quantification by MaxQuant software. The ratios (USP14KO vs WT) appearing in multiple replicates were averaged by arithmetic mean.

Supplementary Data 2.

List of significantly up-regulated ubiquitin sites (p< 0.05, ratio average≥ 1.2) and quantified ubiquitin sites. NA: Not available for identification or quantification by MaxQuant software. The ratios (USP14KO vs WT) appearing in multiple replicates were averaged by arithmetic mean.

Supplementary Data 3.

List of Gene Ontology analysis, KEGG analysis and MCODE cluster results of significantly up-regulated and down-regulated proteins.

Supplementary Data 4.

List of Gene Ontology analysis and KEGG analysis result of significantly up-regulated ubiquitin sites.

Supplementary Data 5.

List of proteins identified in the sample from Flag-USP14 overexpress and Flag-con 293T cells, as well as potential USP14 interacting proteins. Proteins with a five-fold change of the Mascot emPAI score between Flag-USP14 overexpress and Flag-con 293T cells and identified in at least two of the three biological replicates were considered significant. NA: Not identified by Mascot software with an ion score of at least 20.

Supplementary Data 6.

List of overlap among interactome, ubiquitinome and proteome dataset, and ubiquitination level up-regulated but proteome level unchanged USP14 interacting proteins.

Supplementary Data 7.

The clinical characteristics of study subjects.
